# Obesity and high myopia in children and adolescents: Korea National Health and Nutrition Examination Survey

**DOI:** 10.1371/journal.pone.0265317

**Published:** 2022-03-25

**Authors:** Sami Lee, Haeng-Jin Lee, Kyoung Geun Lee, Jihan Kim

**Affiliations:** 1 Department of Family Medicine, Chungnam National University Sejong Hospital, Sejong, Republic of Korea; 2 Research Institute of Clinical Medicine of Jeonbuk National Univeristy-Biomedical Research Institute of Jeonbuk National University Hospital, Jeonju, Republic of Korea; 3 Department of Ophthalmology, Jeonbuk National University College of Medicine, Jeonju, Republic of Korea; 4 Geumnam Public Health Center, Sejong, Republic of Korea; 5 Department of Family Medicine, Sejong Trinium Woman’s Hospital, Sejong, Republic of Korea; 6 Department of Family Medicine, Research institute for Medical Science, Chungnam National University School of Medicine, Daejeon, Republic of Korea; University of Zurich, SWITZERLAND

## Abstract

**Purpose:**

The prevalence of both obesity and myopia are increasing in Korean children and adolescents. The purpose of this study is to examine the impact of obesity on the prevalence of myopia in Korean children and adolescents.

**Methods:**

This study used the data of a nationally representative cross-sectional survey, the Korea National Health and Nutrition Examination Survey (KNHANES) VII conducted from 2016 to 2018. Of the 1237 children and adolescents aged 5–18 years who participated in the KNHANES VII and underwent ophthalmologic examinations for the survey, 1114 were selected for review, excluding those whose data on refractive error, family history of myopia, or waist circumference were missing. Body mass index (BMI) was classified into four groups: underweight (< 5th percentile), normal weight (≥ 5th percentile, < 85th percentile), overweight (≥ 85th percentile, < 95th percentile), and obese (≥ 95th percentile). Myopia was defined by the level of refractive error ≤ -0.5 diopters (D) and classified as mild (≤ -0.5 D, > -3.0 D), moderate (≤ -3.0 D, > -6.0 D), or high (≤ -6.0 D) myopia. The relationship between BMI and myopia was analyzed using complex sample logistic regression. Age and family history were corrected followed by an analysis of the odds ratios.

**Results:**

Compared to those with normal weights (controls), being underweight, overweight, or obese showed no significant odds of developing mild and moderate myopia. Conversely, when compared with that of controls, the odds ratio of developing high myopia in the underweight, overweight, and obese groups was 0.77 (95% CI, 0.22–2.65), 1.37 (95% CI, 0.51–3.66), and 3.77 (95% CI, 1.98–7.16), respectively. Furthermore, in a separate analysis by sex and BMI, the odds ratio of developing high myopia was 2.84 (95% CI, 1.10–7.35) in boys with obesity and 4.23 (95% CI,1.19–15.09) and 5.04 (95% CI,1.77–14.34) in overweight and obese girls, respectively.

**Conclusions:**

An association exists between obesity in childhood and adolescence and high myopia. Being overweight in girls was also found to be associated with high myopia. Thus, efforts to maintain a healthy weight during childhood and adolescence are of great importance.

## Introduction

Childhood and adolescent obesity have been on the rise in recent years due to reduced energy output from convenient living environments, low levels of physical activity, and increased caloric intake [1]. Globally, it was estimated that over 39 million children under 5 years of age were overweight or obese in 2020 and over 340 million children and adolescents aged 5–19 years were overweight or obese in 2016 [2]. In Korea, the prevalence of obesity among adolescents was 5.9% in 2006 but increased to 11.1% in 2019 [3].

One of the most important developments during childhood and adolescence is the vision. Vision development begins after birth and continues until the age of 13 years, with most children reaching the full development between 6 and 8 years of age. Onset of conditions such as refractive error or strabismus may occur during vision development, which, without proper treatment, can lead to amblyopia in adulthood. Myopia, a type of refractive error, is especially important because of its high prevalence and increased risk of other eye diseases such as macular degeneration and retinal detachment [4, 5]. Myopia is a condition in which the cornea or lens is too curved or the eyeball has become too long; hence, the images focus in front of the retina [6]. Approximately 34% of the global population had myopia in 2020 [7]. The prevalence of myopia is slightly higher among Koreans (approximately 54%), across all age groups, with a particularly higher prevalence of 78.8% among children and adolescents aged 12 to 18 years [8, 9].

Myopia is a multifactorial disease, although the contributing genetic and environmental risk factors have been studied [7], the association between obesity and myopia is not well understood. In particular, it is unclear whether childhood or adolescent obesity contributes to the onset of myopia in children and adolescents. Recent studies have suggested that insulin resistance may contribute to the ocular axial length, which in turn results in myopia [10, 11]. In addition, insulin resistance promotes the secretion of Insulin-like growth factor 1 (IGF-1), which also results in axial elongation. Obesity is associated with a higher risk of developing insulin resistance, resulting in an increase in both insulin and IGF levels in the blood. As such, this study aimed to examine the effect of childhood and adolescent obesity on myopia based on the Korea National Health and Nutrition Examination Survey (KNHANES) data.

## Materials and methods

### Data extraction and research ethics

This study was based on data from the KNHANES VII conducted from 2016 to 2018 by the Korea Disease Control and Prevention Agency. The KNHANES, a nationwide, population-based, and health examination and survey on the health status and behavior, including the prevalence of chronic diseases, food intake, and nutritional status of Koreans, is conducted in accordance with Article 16 of the National Health Promotion Act. These are third party data of which not owned or collected by the authors. The acquired data has been kept open to the public by the Korea Center for Disease Control and Prevention; others would be able to access these data in the same manner as the authors. Authors did not have any special access privileges that others would not have. The KNHANES was approved by the Research Ethics Review Committee of the Korea Disease Control and Prevention Agency (Ethics Approval Code: 2018-01-03-P-A) [12]. The study protocol was approved for waiver of review by the Institutional Review Board of Jeonbuk National University Hospital in South Korea (No: 2020-06-005) and followed the tenets of the Declaration of Helsinki.

### Study participants

**Fig 1:**
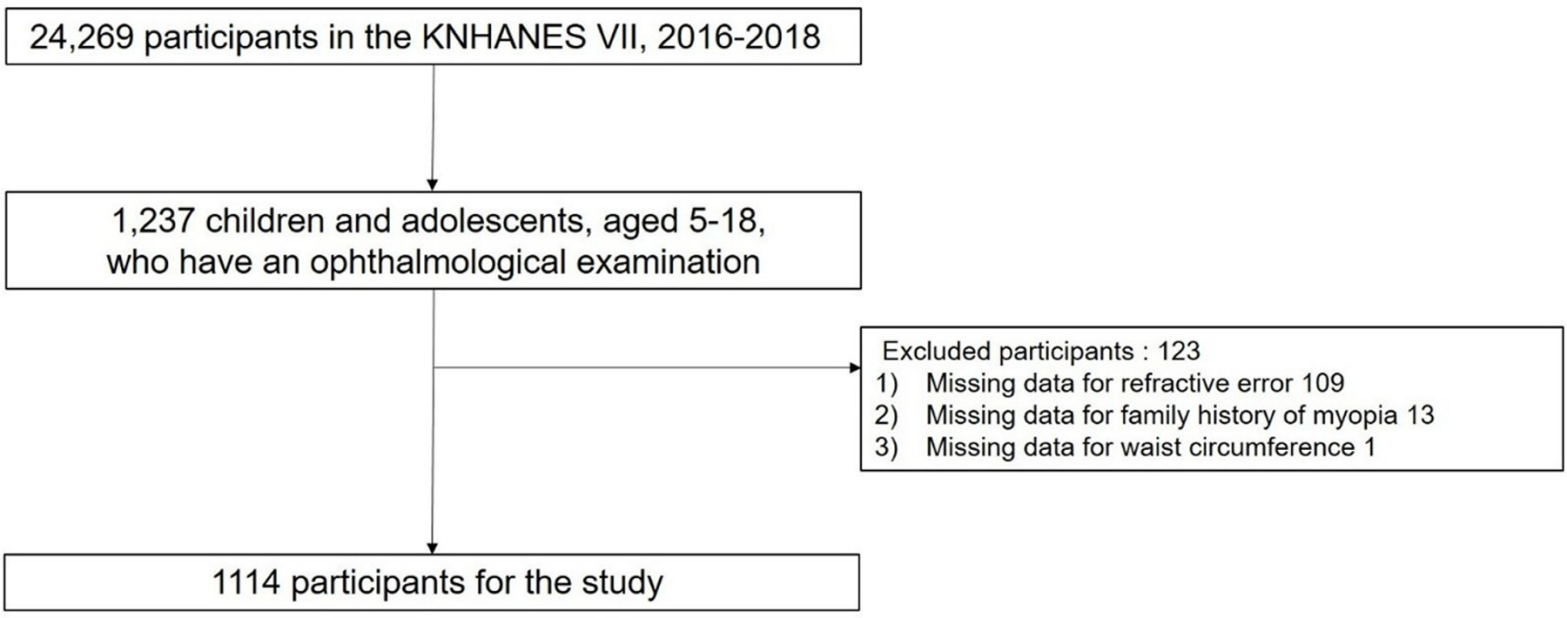
Study population for the study. Of total of 24269 individuals participated in KNHANES VII, 1114 were included in the analysis.

A total of 24269 individuals participated in KNHANES VII. Among the participants, there were 1237 children and adolescents aged 5–18 years who underwent ophthalmologic examination; of these, 1114 were included in the analysis. Those with missing data on refractive error, family history of myopia, and waist circumference were excluded (Fig 1).

### Body measurements and survey variables

The degree of refractive error was measured by noncycloplegic refraction using an autorefractor (KR-8800, Topcon, Tokyo, Japan) in both eyes. Refractive error was analyzed using the mean of the spherical equivalent (SE) refractive error (spherical lens + ½ cylindrical lens) in both eyes.

Body mass index (BMI, in kg/m^2^) was calculated by dividing the body weight by height squared. Height(in cm) was measured using a stadiometer (Seca 225, Germany), while weight (in kg) was measured using a scale (GL-6000-20, G-tech, Korea). Height and weight were recorded to one decimal place. Waist circumference (in cm) was measured from the midpoint between the last lower rib and upper iliac ridge with the participant in a comfortable state with both arms down, upon exhalation, using a tape measure (Seca 200, Germany).

Based on the responses to parental history of myopia (coded as yes, no, unsure, not applicable [children aged under 5 or over 19], or no response); those classified as unsure, not applicable, or no response were excluded, leaving those classified as ‘yes’ or ‘no’. Time spent on near work activities (computer use and reading in the past year) was determined based on responses indicating the average daily time (≤ 1 h, 1–2 h, 3 h, ≥ 4 hours) spent on near work activities. For household income, parental income was classified into quartiles (Q1, Q2, Q3, and Q4) in ascending order.

### Definition of obesity and refraction

Childhood and adolescence obesity were defined according to clinical guidelines as follows: underweight (< 5th percentile), normal weight (≥ 5th percentile, < 85th percentile), overweight (≥ 85th percentile, < 95th percentile), and obese (≥ 95th percentile) [13]. Myopia was defined by the degree of refraction < -0.5 diopters (D) [14]. More specifically, myopia was classified as mild (≤ -0.5 D, > -3.0 D), moderate (≤ -3.0 D, > -6.0 D), or high (≤ -6.0 D) myopia [15, 16].

### Statistical analysis

In analyzing KNHANES data, a two-step stratified sampling method was applied using the survey district and household as primary and secondary sampling units, respectively. Coverage errors, unequal sampling fraction, and non-response errors of non-participants based on the difference in the number of households and the general population between the time of sampling and the survey time, were taken into consideration in the complex analyses, using the primary sampling unit (PSU), stratification (kstrata), and weight variables [17].

BMI was classified into four groups (underweight, normal, overweight, and obese) and then analyzed comparatively for continuous variables such as mean age, height, weight, and waist circumference using a complex sample general linear model procedure. Categorical variables including sex, degree of refraction, family history of myopia, near-work activity, and household income, were analyzed using complex sample cross analysis (Rao-Scott chi-square test). The relationship between BMI and myopia was analyzed using complex sample logistic regression. During the analysis, age and family history were controlled for in the model. All analyses were performed using the IBM SPSS Statistics for Windows, version 22 (IBM Corp., Armonk, NY, USA), and statistical significance was defined as *P* < 0.05.

## Results

### General characteristics of study participants

Of 1114 participants included in this study, 115, 796, 81, and 122 were in the underweight, normal weight, overweight, and obese group, respectively. The average age in each of the groups was 11.6 ± 0.6, 11.9 ± 0.2, 12.6 ± 0.4, and 12.9 ± 0.4 years for the underweight, normal weight, overweight, and obese group, respectively. Furthermore, although the proportion of girls was higher than that of boys in each group, there were no significant differences between the groups. Average height was significantly higher in the overweight and obese groups compared to the normal weight group, at 155.4 ± 2.2 cm and 157. 3± 1.6 cm, respectively. The average weight of the underweight, normal weight, overweight, and obese group of 33.5 ± 1.5 kg, 43.1 ± 0.8 kg, 58.2 ± 2.0 kg, and 68.4 ± 1.9 kg, respectively, differed significantly. The average waist circumference of 55.4 ± 0.8 cm, 63.5 ± 0.4 cm, 76.8 ± 1.1 cm, and 84.7 ± 1.1 cm in the underweight, normal weight, overweight, and obese group, respectively, also differed significantly. Similarly, the average BMI of 15.0 ± 0.2, 18.5 ± 0.1, 23.3 ± 0.3, and 26.9 ± 0.3 in the underweight, normal weight, overweight, and obese group, respectively, differed significantly.

Statistical differences between BMI and refractive error were observed in each group. For high myopia especially, the predictive value (%) in the underweight, normal weight, overweight, and obese group was 5.3 (3.0)%, 6.6 (1.1)%, 9.9 (3.9)%, and 20.6 (4.4)%, respectively. Differences in the predictive value for each group, by BMI, in levels of near-work activities were also statistically significant. On the other hand, no differences were observed between groups according to BMI, family history, or household income (Table 1).

**Table 1:** Characteristics of the subjects according to BMI percentile.

	Low	Normal	Overweight	Obesity	
	<5 percentile	≥5 <85 percentile	≥5 <95 percentile	≥5 percentile	P value
	(N = 115)	(N = 796)	(N = 81)	(N = 122)	
**Age (years)**	11.6±0.6	11.9±0.2	12.6±0.4	12.9±0.4*	
**Sex**					0.351
** Men**	56.0 (4.9)	50.9 (2.1)	61.1 (5.6)	54.5 (5.5)	
** Women**	44.0 (4.9)	49.1 (2.1)	38.9 (5.6)	45.5 (5.5)	
**Height (cm)**	145.8±2.5	149.2±1.0	155.4±2.2^†^	157.3±1.6^‡^	
**Weight (kg)**	33.5±1.5^‡^	43.1±0.8	58.2±2.0^‡^	68.4±1.9^‡^	
**W.C (cm)**	55.4±0.8^‡^	63.5±0.4	76.8±1.1^‡^	84.7±1.1^‡^	
**BMI (kg/㎡)**	15.0±0.2^‡^	18.5±0.1	23.3±0.3^‡^	26.9±0.3^‡^	
**Refractive errors**					0.004
** >-0.5 (D)**	38.7 (5.5)	30.7 (2.1)	21.3 (5.7)	22.2 (4.8)	
** >-3.0, ≤-0.5**	38.5 (5.3)	39.8 (1.9)	40.4 (7.5)	32.1 (4.4)	
** >-6.0, ≤-3.0**	17.5 (4.2)	22.9 (1.9)	28.4 (5.8)	25.1 (4.6)	
** ≤-6.0**	5.3 (3.0)	6.6 (1.1)	9.9 (3.9)	20.6 (4.4)	
**Parent myopia**					0.436
** Yes**	75.0 (4.9)	70.3 (2.2)	65.4 (5.9)	64.5 (5.9)	
** No**	25.0 (4.9)	29.7 (2.2)	34.6 (5.9)	35.5 (5.9)	
**Near work**					0.020
** ≤1 (hour/day)**	16.3 (3.6)	11.1 (1.4)	7.6 (2.3)	3.1 (1.7)	
** 1–2**	33.4 (5.0)	32.2 (2.0)	27.5 (4.9)	22.3 (4.3)	
** 3**	21.1 (4.8)	19.8 (1.6)	24.6 (5.0)	28.6 (5.1)	
** ≥4**	29.3 (5.4)	36.9 (1.9)	37.1 (6.4)	46.4 (5.7)	
**Income**					0.059
** Q1**	7.3 (2.7)	8.5 (1.3)	13.9 (4.3)	18.8 (5.0)	
** Q2**	18.4 (4.0)	25.9 (2.7)	22.1 (6.0)	26.9 (5.8)	
** Q3**	37.3 (6.4)	32.1 (2.7)	22.0 (4.9)	29.8 (5.1)	
** Q4**	37.0 (6.6)	33.5 (3.6)	42.0 (7.4)	24.5 (5.1)	

All analyses used integrated weight values.

Values are presented as estimated % (SD) or mean ±SD.

*, ^†^ and ^‡^ are presented p<0.05, p<0.01 and p<0.001 compared to normal BMI group by complex samples general linear model.

Group variables are analyzed by Rao-Scott chi-square test.

### Proportion of mild, moderate, and high myopia by BMI groups

**Fig 2:**
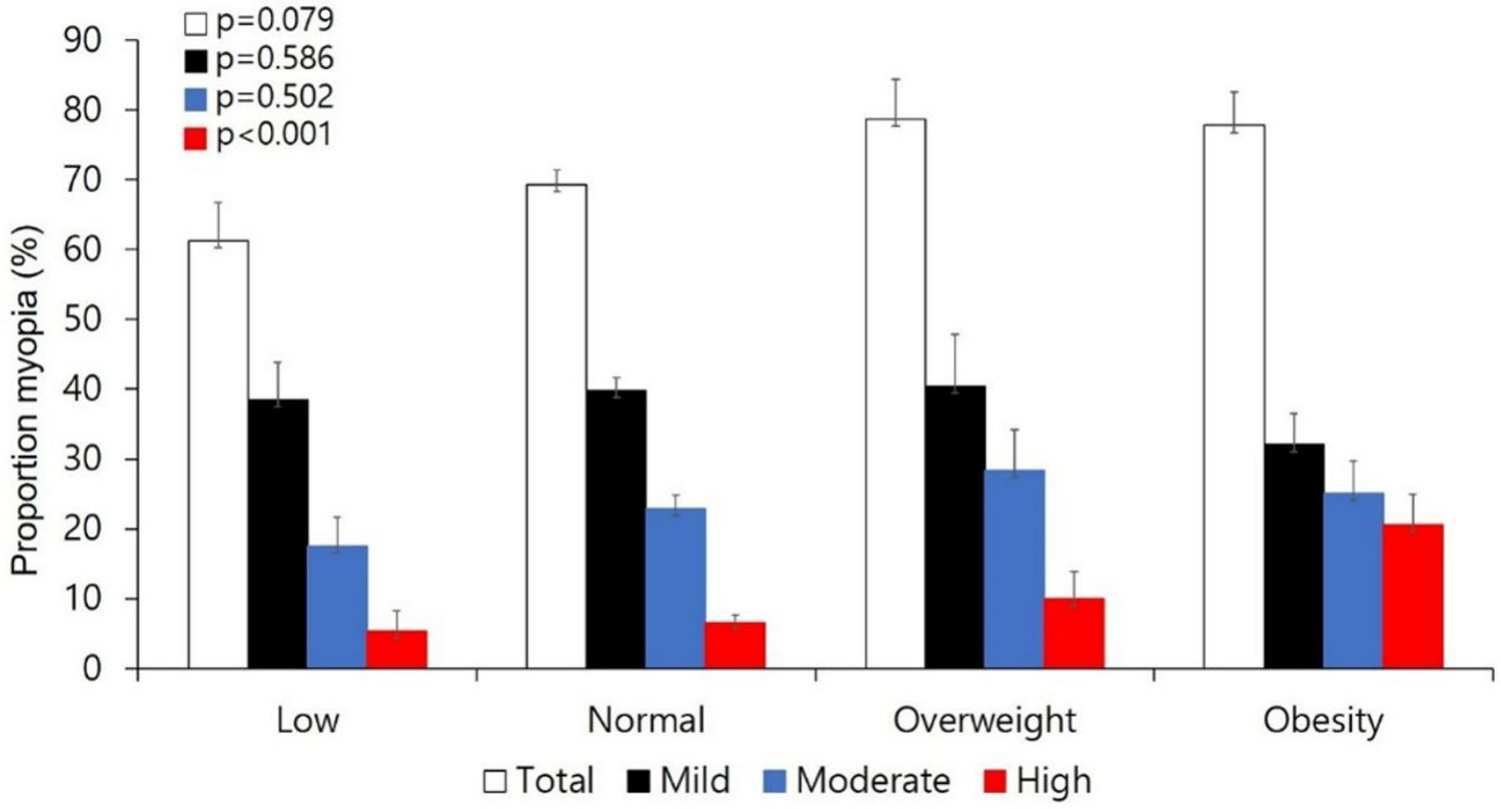
Estimated proportion of mild, moderate and high myopia according to BMI percentile using Rao-Scott chi-square test. The estimated proportion of those with high myopia in the underweight, normal weight, overweight, and obese groups were 5.3%; 6.6%; 9.9%; and 20.6%, respectively (*P* < 0.001).

The comparison by the presence or absence of mild, moderate, or high myopia according to BMI groups revealed that an estimated 61.3% (±5.5%); 69.3 (±2.1%); 78.7 (±5.7%); and 77.8% (±4.8%) in the underweight, normal weight, overweight, and obese group had myopia, respectively; but with no significant differences between groups (*P* = 0.079). On the other hand, significant differences were observed between groups in the severity of myopia, in particular high myopia. The estimated proportion of those with high myopia in the underweight, normal weight, overweight, and obese groups were 5.3% (±3.0%); 6.6% (±1.1%); 9.9% (±3.9%); and 20.6% (±4.4%), respectively (*P* < 0.001) (Fig 2).

### Risk analysis of myopia by BMI using complex samples logistic regression

Risk analysis for the severity of myopia by BMI was conducted, controlling for age, parental history, height and near work. The odds ratios for mild myopia in the underweight, overweight, and obese groups compared to the normal weight group were 0.77 (95% confidence interval [CI], 0.39–1.52); 1.25 (0.56–2.82); and 0.88 (0.49–1.58), respectively. The odds ratios for moderate myopia in the underweight, overweight, and obese groups compared with that in the normal weight group was 0.72 (0.38–1.37); 1.22 (0.57–2.59); and 0.82 (0.46–1.49), respectively. For high myopia, the odds ratios for the underweight, overweight, and obese groups compared with that in the normal weight group was 0.77 (0.22–2.65); 1.37 (0.51–3.66); and 3.77 (1.98–7.16), respectively (Table 2).

**Table 2:** Odds ratio for mild, moderate and high myopia according to BMI percentile in multivariable logistic regression analysis.

	Mild myopia	Moderate myopia	High myopia
	OR (95% CI)	OR (95% CI)	OR (95% CI)
**Low (<5 percentile)**	0.77 (0.39–1.52)	0.72 (0.38–1.37)	0.77 (0.22–2.65)
**Normal (≥5, <85 percentile)**	Reference	Reference	Reference
**Overweight (≥85, <95 percentile)**	1.25 (0.56–2.82)	1.22 (0.57–2.59)	1.37 (0.51–3.66)
**Obesity (≥95 percentile)**	0.88 (0.49–1.58)	0.82 (0.46–1.49)	3.77 (1.98–7.16)
**(Adjusting factor)**			
**Parent myopia**	1.61 (1.12–2.31)	1.67 (1.09–2.56)	2.99 (1.47–6.09)
**Age**	1.05 (0.93–1.18)	1.13 (1.03–1.24)	1.15 (1.01–1.31)
**Height**	1.05 (1.03–1.08)	1.02 (1.00–1.04)	1.05 (1.02–1.07)
**Near work** *	1.10 (0.68–1.79)	2.92 (1.24–6.86)	0.75 (0.19–3.02)

All analyses used integrated weight values.

* presented >1 hour/day.

In a separate multifactorial regression analysis of high myopia based on BMI adjusted by sex, overweight boys had a 2.84-fold (95% CI, 1.10–7.35) higher odds of having high myopia, while overweight and obese girls had a 4.23-fold (95% CI, 1.19–15.09) and 5.04-fold (95% CI, 1.77–14.34) higher odds, respectively, of having high myopia (Table 3).

**Table 3:** Odds ratio of high myopia according to BMI percentile and sex in multivariable logistic regression analysis.

	High myopia	
	OR (95% CI)	
	Male	Female
**Low (<5 percentile)**	0.28 (0.05–1.46)	1.43 (0.33–6.23)
**Normal (≥5, <85 percentile)**	Reference	Reference
**Overweight (≥85, <95 percentile)**	0.40 (0.06–2.74)	4.23 (1.19–15.09)
**Obesity (≥95 percentile)**	2.84 (1.10–7.35)	5.04 (1.77–14.34)
**(Adjusting factor)**		
**Parent myopia**	1.56 (0.63–3.85)	10.65 (1.97–57.63)
**Age**	1.15 (0.94–1.39)	1.09 (0.89–1.33)
**Height**	1.07 (1.03–1.11)	1.06 (1.01–1.10)
Near work*	0.76 (0.08–7.31)	0.61 (0.12–3.02)

All analyses used integrated weight values.

* presented >1 hour/day.

## Discussion

This study examined whether childhood and adolescence obesity was associated with myopia, and results indicate an odds ratio 3.77 times higher than those with normal weight in developing high myopia among obese children and adolescents; 2.84 and 5.04 times higher odds occurred in males and females, respectively. Furthermore, even in the overweight category, the females had an odds ratio of 4.23 times higher than that of those with the normal weight.

According to previous research, obesity in schoolchildren increased the risk of myopia 2.7 times [18]. Moreover, a Dutch study on children aged 6 years demonstrated an association between myopia and high BMI [19]. However, the classification of myopia used in the previous study is different from the classification of myopia in our study. These studies defined myopia only as <-0.5 D refractive error; analyzing the mild, moderate, and high myopia groups inclusively. In our study, myopia was more specifically classified as mild, moderate, and high myopia. Since there was no separate analysis by degree of refractive error, it is difficult to compare their findings directly with the findings of this study.

A study conducted in a Korean population reported that the risk of developing high or moderate myopia is 1.03 times greater in individuals with a higher BMI [20]. A study by Kim et al. [21] aimed to identify an association between refraction errors and potential risk factors for myopia; one such risk factor was BMI. Although they showed that a higher BMI may be associated with high myopia, the BMI was not based on the criteria for diagnosing obesity in children and adolescents and was used as a continuous variable in the analysis of the associated risk factors. In our study, children and adolescents were organized according to accurate obesity diagnostic criteria to determine the influence of obesity on myopia in each BMI group; obesity was classified based on BMI, and the relationship with myopia was investigated. In the case of children and adolescents, it is necessary to conduct a comparative analysis of myopia with the BMI group because BMI, the diagnostic criteria for obesity, varies according to sex or age. Therefore, the relationship between obesity and myopia in our study was analyzed in detail using a more accurate definition of obesity based on BMI by age and sex. The advantage of the current study design is its focus on obesity and the association between myopia and obesity.

The exact causal relationship between obesity and myopia is not well known. Although obesity generally causes a variety of complications, one factor that may be associated with myopia is insulin resistance. Insulin resistance is one of the most common biochemical phenomena observed in obesity. Postprandial or fasting hyperglycemia caused by delayed initial insulin secretion is characteristic of insulin resistance and has been reported to occur in 15% to 20% of obese children [22]. According to a study conducted in those without diabetes, insulin secretion is suppressed under hyperglycemic conditions, which contributes to the thickening of the lens and an anterior shift of the anterior pole; worsening the myopia [23]. Furthermore, an increase in blood insulin levels is known to promote the secretion of Insulin-like growth factor 1 (IGF-1), which promotes cell growth and differentiation, leading to the axial elongation characteristic of myopia in the eye [10]. Although an accurate comparison and analysis are difficult, since this study did not examine the relationship between insulin resistance and myopia, it is likely that insulin resistance is the most relevant for predicting the causal relationship between obesity and myopia.

Myopia is a multifactorial disease that involves both genetic and environmental factors. Although not many studies have identified the causal relationship, the most recognized environmental factors thus far have been, near-work and outdoor activities [24, 25]. The correlation between near-work and BMI [26] is supported in this present study where differences in the duration spent performing near-work activities were observed between BMI groups. Considering the complex etiology of myopia, not only genetic and environmental factors but also body stature may associate with myopia. According to findings from previous study, tall girls have longer axial lengths, longer vitreous chambers, thinner lenses, and flatter corneas than boys. Moreover, boys are characteristically more hyperopic at higher body weights [27]. Gunes et al. [28] reported that retrobulbar fat is limited by the orbital space, preventing expansion, unlike other fat tissue deposits in the body. Therefore, obese individuals tend to have more hyperopic vision and shorter vitreous chambers. However, there is no evidence of an association between connective tissue disease and the development of high myopia.

Another study, which included data from a survey of young adult Korean men who underwent physical examinations performed by the Korean Military Manpower Administration from 2009 to 2013, reported that taller and leaner men were more likely to have high myopia [29]. There are a few key differences between our study and the study design in Lee et al. [29] They only included men aged 18–35 years; most of whom were aged 19 years (approximately 97%). However, we included both male and female children under the age of 19 years. These differences might explain the contradictory results, highlighting the need for additional research to be performed on children, as their growth parameters (such as height and weight) change each year.

The strengths of this study include a well-constructed design, a large dataset, and the nationwide representativeness of the original survey. Nevertheless, there are a few limitations to generalizing the findings of this study. First, this was a cross-sectional study, making it difficult to accurately identify causality. Second, this study did not involve ophthalmic tests including factors characterizing myopia, other than the degree of refraction. In addition, the refractive error in KNHANES was measured without cycloplegia, which may have resulted in an overestimation of the prevalence of myopia. Cycloplegic refraction is the most accurate method for analyzing refractive errors in children, but it is difficult to perform in a large population. This is a common limitation in studies on refractive errors using the KNHANES and United Stated NHANES datasets. Despite this limitation, many studies have provided meaningful results; however, in future studies, analysis of the refractive error obtained using cycloplegic refraction must be considered. Third, although the risk of high myopia was found to be higher in overweight and obese girls, the confidence intervals observed in these groups were broad. This limitation may be owing to the small sample size; therefore, a future study with a larger sample size should be performed.

In conclusion, the findings of this study are significant in that it identified an association of childhood and adolescence obesity with high myopia because of the analysis of the national KNHANES data involving a large number of participants. Although additional research is needed, efforts to maintain a healthy weight among children and adolescents will be necessary to decrease the risk of high myopia.
